# Risk-adjusted/neuroprotective care services in the NICU: the elemental role of the neonatal therapist (OT, PT, SLP)

**DOI:** 10.1038/s41372-020-0597-1

**Published:** 2020-01-28

**Authors:** Jenene W. Craig, Catherine R. Smith

**Affiliations:** 1 Infant and Early Childhood Development (IECD) PhD Program, Fielding Graduate University, 2020 De la Vina Street, Santa Barbara, CA 93105 USA; 2 Department of Physical Therapy, University of Tennessee at Chattanooga, 615 McCallie Avenue, Chattanooga, TN 37403 USA

**Keywords:** Rehabilitation, Health occupations

## Abstract

Infants admitted to neonatal intensive care units (NICU) require carefully designed risk-adjusted management encompassing a broad spectrum of neonatal subgroups. Key components of an optimal neuroprotective healing NICU environment are presented to support consistent quality of care delivery across NICU settings and levels of care. This article presents a perspective on the role of neonatal therapists—occupational therapists, physical therapists, and speech–language pathologists—in the provision of elemental risk-adjusted neuroprotective care services. In alignment with professional organization competency recommendations from these disciplines, a broad overview of neonatal therapy services is described. Recognizing the staffing budget as one of the more difficult challenges hospital department leaders face, the authors present a formula-based approach to address staff allocations for neonatal therapists working in NICU settings. The article has been reviewed and endorsed by the National Association of Neonatal Therapists, National Association of Neonatal Nurses, and the National Perinatal Association.

## Introduction

Infants requiring neonatal intensive care are a particularly vulnerable population secondary to prematurity and/or significant medical conditions. Risk-adjusted care considers the broad spectrum of medical, neurologic, developmental, and psychosocial outcomes experienced by neonatal subgroups [1]. The effectiveness of providing the highest level of care to support family-centered, holistic developmental care services to improve short- and long-term outcomes for preterm and medically fragile neonates is well documented in the literature [2–9]. This has resulted in a standard of care for implementation of developmental care procedures in patient management practices in many neonatal intensive care units (NICUs) in the United States and around the world. However, the elemental components needed to create an optimal neuroprotective healing environment for infants in the NICU lack the requisite standardization recommendations to ensure consistent quality of care delivery across NICU settings and NICU levels of care. A 2017 joint position statement from the Canadian Association of Neonatal Nurses, Canadian Association of Perinatal and Women’s Health Nurses, National Association of Neonatal Nurses (NANN), and Council of International Neonatal Nurses addressed this concern by detailing guidelines for the institutional implementation of developmental neuroprotective care in the NICU [10, 11]. Inclusion of neonatal therapists (NTs) as essential components of a comprehensive preventive model of developmental care in the joint position statement acknowledged the critical contribution of the therapy disciplines to developmental care service design and delivery in the NICU [11, p. 65]. Relatedly, the current article presents a perspective on the role of NTs—occupational therapists (OT), physical therapists (PT), and speech–language pathologists (SLP)—in the provision of elemental risk-adjusted, neuroprotective care services in the NICU.

## Background

Neonatal therapy encompasses the art and science of integrating typical development of the infant and family into the environment of the NICU [12–16]. Incorporating theories and scopes of practice from the respective disciplines of occupational therapy, physical therapy, and speech-language pathology, neonatal therapy requires advanced knowledge of the diagnoses and medical interventions inherent to the NICU setting in order to provide safe and effective assessment, planning, and treatment [17]. While the provision of developmental neuroprotective care is a fundamental neonatal nursing responsibility, the five core measures included in the 2011 NANN guidelines serve as imperatives that an optimal neuroprotective environment requires the coordination of care with disciplines of medicine and nursing, including the scope of practice of NTs [18–23]. Skilled neonatal therapy competencies support preventative intervention from birth to enhance physiologic function and neurostructural development of the infant with benefits extending to all stakeholders including the infant, family, healthcare community, and provider networks [11, 24–36].

The Universe of Developmental Care (UDC) Model provides a useful framework to underscore the value of including neonatal therapy as an elemental component of quality service delivery in the NICU [37]. Recognizing the foundational interdependence of a shared surface interface when defining developmental care, the UDC model represents the impact of all body systems and the environment on brain development. Serving as an extension of the Synactive Theory proposed originally by Dr Heidelise Als, UDC purports that “all interactions begin at the organism–environment interface,” with the interface between the infant’s body and the environment serving as the tangible link between the person and all elements of the micro- and macroenvironment [38, 39, p. 146]. Accurate identification of both antecedents and consequences of consistent neuroprotective care formulates the basis to better understand the impact of the organism–environment interface as crucial to the delivery of quality care in the NICU. NTs are integral to the creation of a sensitive transactional interface through their understanding of sensory and environmental factors impacting critical elements of development.

### Elemental roles of the neonatal therapist

Defining the necessary components required to provide risk-adjusted age-appropriate neonatal care for complex and critically ill infants will facilitate implementation of standardized care practices consistent with the central tenets of developmental care philosophy and the demonstrable effect on perinatal outcomes. The American Occupational Therapy Association (AOTA), American Physical Therapy Association (APTA), American Speech–Language–Hearing Association (ASHA) define the NICU as a specialized practice setting due to the medical and developmental fragility of the infants, the vulnerable emotional status of the families, and the intricacy of medical, cultural, and social factors that impact the family-infant unit [20–23, 40]. NTs apply knowledge of neonatal medical conditions, intensive care equipment, preterm infant development and necessary handling precautions, and family system dynamics to contribute to the development of a collaborative management plan that promotes age-appropriate infant neurobehavioral organization and interactions. Interventions provided by NTs optimize long-term development, prevent adverse sequelae, nurture the infant-family dyad, and support education needs of the family and NICU team [12].

In accordance with the American Academy of Pediatrics (AAP), NTs contribute to the provision of care in each of the four levels of nurseries as defined below [1]:

- Level I well newborn nurseries (WNNs) provide basic routine care including state-mandated newborn assessment, management of minor medical problems, and screenings and medical stabilization to prepare for transport when needed for low risk, full-term, and late-preterm infants born 35–37 weeks gestational age.
- Level II special care nurseries (SCN) provide intermediate level care for moderately preterm infants born at greater than or equal to 32 weeks gestational age with birth weight greater than 1500 g or management of full-term infants requiring careful postnatal monitoring, IV antibiotics, or short-term respiratory support.
- Level III or IV NICUs provide a full range of subspecialty staffing with advanced diagnostic imaging and respiratory support equipment adequate to provide the highest-level intensive care services for critically ill infants of all viable gestational ages. Level IV NICUs additionally provide requisite surgical subspecialties for surgical repair of complex conditions as well as a full range capacity of respiratory support including extracorporeal mechanical oxygenation [1].

As essential members of the SCNs and NICU teams, NTs provide family-centered neuroprotective care services to infants at increased risk for developmental compromise and their families. Infants with lower medical acuity admitted to the NBN (WNN) or Level II SCN who benefit from neonatal therapy services include—but are not limited to—neonates born at 32 weeks gestation or older, neonates who have been exposed prenatally to recreational or prescription drugs, infants with birth injuries/anomalies, late preterm infants, infants born to young teen parents or mothers with inadequate prenatal care. Per AAP recommendations, all infants with higher medical acuity at increased risk for developmental compromise admitted to Level III or IV NICUs merit support through comprehensive services by NTs [1]. Neonatal therapy is optimally provided through an integrated collaborative-care model.

The provision of therapy services in the NICU requires specialized orientation, training, continuing education, and mentoring. Discipline-specific competencies exist that support the presence of all three disciplines in the NICU to best address the unique needs of the infants and their families in this complex treatment environment. While each NT (OT, PT, and SLP) brings neuroprotective developmental support tasks that fall specifically within the scope of practice of their discipline, there are foundational neonatal therapy service skills included within the scope of practice across all three disciplines of OT, PT, and SLP. While the presence of more than one neonatal therapy discipline may offer the best practice, staffing patterns may vary among hospitals based on a combination of availability of therapy staff members who possess the requisite advanced discipline-specific competencies to provide the specialized services required in the NICU practice setting and service needs. The competencies common to all three discipline’s scope of practice are optimally provided in a manner that reflects the discipline-specific perspective of each professional while avoiding redundant overlap of services.

To clarify differences and commonalities among the scopes of practice of the therapy disciplines providing neonatal therapy services, OT, PT, and SLP benefit from their state licensure scope of practice guidelines as well as advanced neonatal therapy competency recommendations endorsed by the discipline-specific professional organizations (AOTA, APTA, and ASHA). In addition, the National Association of Neonatal Therapists (NANT) empaneled the NANT Professional Collaborative (NPC) to identify the common knowledge, skills, and abilities needed for a NT to practice safely and competently in the NICU regardless of his or her professional affiliation. The NPC is a multidisciplinary professional task force of NTs (OT, PT, and SLP) established to advise and assist in defining, creating and reviewing emerging standards, practices, and guidelines. The NPC has defined a Neonatal Therapy Core Scope of Practice^©^, which highlights the ‘shared’ common ground of all three disciplines but is not intended to represent the unique aspects of service inherent to each individual discipline [41]. It is crucial to consider that evaluation and intervention practices of each neonatal therapy discipline (OT, PT, and SLP) vary based on diagnosis or problem, scope of practice, and state regulations. Reference to associated national professional organization (AOTA, APTA, ASHA, and NANT) for discipline-specific skills, knowledge, and roles will be necessary. However, it is imperative that each therapist considered for work in the NICU possess the fundamental baseline cross-discipline knowledge. With acknowledgment of the discipline-specific lens, Table 1 provides details on foundational and essential knowledge areas of the NT that contribute to evaluation and intervention in the NICU [41, 42]. The underlying assumptions regarding assessment and intervention include that:

1. Intervention requires continuous assessment.
2. Assessment includes pre/during/postintervention.
3. Assessment can be formal and/or informal and includes observational assessment.
4. The development of subsystems is connected one to another.
5. Medical status, diagnoses, age, and medications affect assessment/intervention.

**Table 1:** Knowledge areas for evaluation and intervention in the neonatal intensive care unit.

Knowledge area	Evaluation/intervention
Environment (micro-, macro)- including equipment	NT^a^ identifies how the environment affects the infant, determines if environment appropriately matches each infant’s age-specific/risk-adjusted/individual needs, and modifies/adapts environmental affordances according to age-appropriate abilities.
Neurodevelopment-immaturity secondary to preterm or late preterm birth and iatrogenic risks/impact affecting	NT uses assessments and implements interventions that match each infant’s neurodevelopmental needs and sensory input/motor output thresholds from birth through discharge as indicated.
• Neurobehavioral system	NT interprets quality of neurobehavioral output as it relates to environmental input. Generates or formulates an intervention plan that supports the infant’s capacity/skill development in: • autonomic, • motor, • state, • attention, and • self-regulation.
• Neuromotor system	NT interprets quality of neuromotor system as influenced by environmental affordances. Generates or formulates an intervention plan that supports each of the following with age-appropriate interventions: • Neurodevelopmental positioning and handling for caregiving, rest, and recovery • Movement pattern development • Reflex development • Muscle tone development/changes • Compromise following insult (e.g., hypoxic ischemic encephalopathy, IVH, PVL, dysgenesis of corpus callosum)
• Musculoskeletal system	NT interprets quality of musculoskeletal system support and function as it relates to environmental input. Generates or formulates an intervention plan that supports the infant’s development of: • Posture and alignment development • Antigravity movements and symmetric strength development, • Physiological tolerance of activity • Management of orthopedic anomalies (e.g., brachial plexus injury, club foot, spina bifida, etc.) • Prevention of iatrogenic deformities
• Sensory system ○ Tactile ○ Proprioceptive ○ Vestibular ○ Gustatory ○ Olfactory ○ Auditory ○ Visual	NT Interprets quality of sensory input as it relates to environmental input. Generates or formulates an intervention plan that supports sensory system development at micro- and macro level and promotes protection of sensory system components during age-appropriate activities: • Sensory integration capabilities, and • Progression of sequential sensory system development.
• Aerodigestive system	Feeding assessment must be in the scope of practice of the individual discipline.
Successful transition to oral feeding requires the infant integrate skills from multiple systems and adjust for existing comorbidities	Although not all NT’s may have specialization in oral feeding and/or swallowing, the NT must be able to assess a number of skills that are foundational in order to support oral feeding acquisition. NT promotes protection of aerodigestive system components during age-appropriate activities: • Sensory oral and gustatory integration, • Pre-feeding and safe transition to oral feeding, including breast and bottle feeding, • Autonomic system dysregulation/compromise, • Structural anomalies affecting development or oral feeding/swallowing (e.g., cleft palate, tracheoesophageal fistula, etc.), and Assessment may include clinical bedside and instrumental (e.g., graphic assessment, VFSS, FEES, etc.).
• Pain management	Nonpharmacological pain management interventions.
Family/psychosocial: individual family needs: culture, socioeconomic, language, financial, etc. (in coordination with nursing and other mental-healthcare providers)	Assess confidence and competence in the following • Bonding and attachment • Psychological support • Cognitive abilities and/or challenges • Early caregiving activities • Transition to home Intervention includes unit-wide and family-specific education and support, and strategies to guide families in early parenting skills by improving confidence and competence in noted assessment areas.

^a^Neonatal therapist.

The Neonatal Therapy Core Scope of Practice^©^ additionally provides foundational definitions of neonatal therapy and subsequent services required to provide risk-adjusted age-appropriate neonatal care for medically fragile infants and families [41]. The NT must have knowledge of medical interventions occurring in tandem with therapeutic interventions and must be skilled in appropriate timing and intensity of interventions within a tenuous environment. Beginning at birth, neonatal therapy promotes optimal long-term developmental outcomes and nurtures infant–parent relationships by addressing the synergistic neurodevelopmental systems foundational to the development of functional skills including the neurobehavioral, neuromotor, neuroendocrine, musculoskeletal, sensory, and psychosocial domains [41].

### Recommendations for neonatal therapy staffing

In alignment with professional organizations (AOTA, APTA, ASHA, and NANT), including the Neonatal Therapy National Certification Board (NTNCB), and in attending to the AAP leveling of neonatal care, the following recommendations are made for neonatal staffing [1, 20, 23, 41 56–59, 61].

#### Level I well newborn nursery (WNN)

For Level I WNNs, therapists may serve a consultative role. For Level I units in hospitals with Levels II–IV NICUs, a process of access to NTs should be established. For hospitals without a NICU, NTs may offer consultative work to the WNN as part of an outside institution to provide in-person consultation, and/or direct patient care as part of a service arrangement between entities. For remote consultation to the Level I WNN, the process should be a formal and established system for consultative input via phone/telemedicine with specialized NT(s) from a level III or IV NICU. The process should establish how to access a trained NT (OT, PT, and SLP) who can be available for remote and/or in-person consultation (preferred) within a timely manner based on acuity to meet the infant and family needs. Consulting services may vary by discipline based on diagnosis or presenting clinical problems, scope of practice, and state regulations depending on the practice patterns of the consulting institution. Examples of presenting problems/diagnoses appropriate for referral for neonatal therapy services in the Level I WNN may include—but are not limited to:

- Neonatal Abstinence Syndrome resulting from prenatal exposure to recreational or prescription drugs.
- Birth injuries or structural anomalies.
- Neuromotor or orthopedic diagnoses.
- Genetic disorders, fetal distress, or neurodevelopmental immaturity affecting safe transition to oral feeding.

#### Level II special care nursery (SCN)

The developmental care needs of infants admitted for intermediate level care to a Level II SCN vary according to degree of prematurity and medical acuity. Serving infants born as young as 32 weeks gestational age, as well as infants requiring short-term respiratory support or recovering from intrauterine drug exposure, the SCN merits careful attention to the delivery of consistent high-quality developmental care. Service delivery models may vary according to number of beds and patient acuity level, however, ensuring availability of neonatal therapy expertize is important in this setting to optimize neurodevelopmental outcomes and prevent iatrogenic complications. For smaller SCNs located in a community hospital that do not have in-house access to neonatal therapy expertize, establishing a formal process for providing consulting services by qualified NTs as described above may be considered. For SCNs that serve as a step-down unit for higher acuity level NICUs in the same facility, staffing should be considered as part of the FTE calculation as stated below. A process to ensure consistent transfer of neuroprotective care principles between units should be implemented.

#### Levels III and IV NICU

Historically neonatal therapy services in NICU Levels III and IV evolved through application of a multidisciplinary rehabilitative/consultative model of care approach. However, the classic rehabilitative/consultative model does not adequately address the complex developmental needs of the preterm or medically fragile neonate. Similar to the benefits documented for primary care nursing staffing patterns, current best practice for intensive care neonatal therapy service delivery emphasizes an expanded integrated model [43, 44]. Focusing on preventative and habilitative interventions delivered in a transdisciplinary manner that protect the infant from unnecessary stress and iatrogenic complications, discipline-specific specialized neonatal therapy interventions are designed to ameliorate prenatal or acquired system compromise of the medically fragile infant [41]. Services are also directed to the staff and primary family caregivers to promote their skills in handling and interacting with an infant who may be vulnerable and medically fragile, even when discharged from the NICU.

Neonatal Therapists working in Levels III–IV NICU settings under an integrated model should have consistent dedicated days/time in the NICU to assess and address age-appropriate patient and family needs, from any viable gestational age through discharge. The AAP Guidelines for Perinatal Care (8th edition) recommends at least one OT or PT with neonatal expertize and at least one individual skilled in evaluation and management of feeding and swallowing disorders in Level III/IV NICUs [1, p. 58]. As an elemental part of the NICU team, NTs provide direct preventive and habilitation services within a neuroprotective framework, and indirect services including medical and/or developmental rounds, staff development, NICU committees, CQI initiatives, family meetings and education delivery, and leadership opportunities. Table 2 provides models of service care according to unit level.

**Table 2:** Models of care delivery.

Unit level	Description	Model
Level I well newborn nursery (NBN)	Formal and established system for consultative input via phone/telemedicine with specialized neonatal therapists from Level III or IV NICU.	Remote consultation
Level II special care nursery	Consultative or ongoing direct patient care. Neonatal therapists may work at an outside institution but provide in-person consultation and/or direct patient care as a part of a service arrangement between entities. Level II nurseries that serve as a step-down unit to a higher acuity NICU would have neonatal therapy staffed consistent with an integrated neonatal therapy model.	In-person services (Integrated neonatal therapy if connected to a higher acuity NICU)
Level III NICU and Level IV regional NICU	Neonatal therapy is an elemental part of the NICU team and neonatal therapists have consistent dedicated day/time frame in the NICU, even if not an entire FTE. They participate in medical and/or developmental rounds, committees, CQI, family meetings, and leadership opportunities within the NICU as appropriate and work within a systematic model of collaborative neuroprotective care based on known risk factors.	Integrated neonatal therapy

#### Neonatal follow-up services

All vulnerable survivors of a complicated perinatal course designated as ‘high risk’ for medical, neurologic, developmental, or psychosocial outcomes following discharge from Levels II to IV care units merit follow-up care to mitigate adverse outcomes associated with their physiologic compromise and to track, record, and analyze medical and neurodevelopmental outcomes. The AAP Guidelines for Perinatal Care, 8th edition, recommends that follow-up services are an ‘essential’ component of Levels III and IV NICU services [1]. Service delivery will vary according to the needs of the infants being monitored, ranging from preventive monitoring of growth and development, management of unresolved medical problems, early identification of delayed developmental progress, monitor for environmental and psychosocial concerns, referral for early intervention, community support and habilitation services, and documentation of long-term outcomes [1]. Neonatal Therapists provide invaluable neonatal follow-up support through active participation in NICU discharge planning, participation in NICU follow-up clinics and through the provision of out-patient services when warranted.

### Neonatal therapy staffing challenges

The staffing budget is one of the most difficult challenges hospital department leaders face in their work as labor consumes the majority of an organization’s financial resources [45, p. 25]. Discussion regarding comprehensive staffing complexities is beyond the current scope of this paper, however, effective staffing plans must consider the balance between quality, safety, staff satisfaction, census variability, and fiscal responsibility. In addition, efforts to provide the most cost-effective and the highest quality patient care must take into account the importance of achieving neonatal therapy staff with the correct skill mix [45]. Given evidence-based findings regarding infant (and family) outcomes related to neuroprotective and developmentally supportive interventions in the NICU and the role of the NT, consideration around more adequate staffing of neonatal therapy must be addressed.

Neonatal care has become technically advanced and requires a sufficient supply of specially trained professionals [46]. The rising number of preterm and multiple births makes it critical to recruit and retain sufficient and appropriate staff to provide quality of care and must include interdisciplinary staffing [46]. A gap in the literature exists regarding staffing of NTs; however, the NANN RN NICU staffing position statement, includes the American Nurses Association consideration of other healthcare professionals in collaborative, interdisciplinary partnership as a core component of staffing the NICU [47, 48, p. 6].

Early therapy services in the NICU that are continued routinely until NICU discharge optimizes outcomes [49, 50]. Borrowing from the nursing literature, skill mix in staffing has additionally been found to result in improved patient outcomes [51]. Relatedly, lower patient-to-nurse ratios are believed to decrease nurse turnover, hospital complications, length of stay, and increase cost savings; however, there is substantial NICU nurse understaffing relative to national guidelines associated with increased risk for very low birth weight (VLBW) infants [52, p. 444]. Similarly, staffing of NTs is also currently variable across units in the United States and nonexistent in many, in spite of ample evidence regarding the benefits of neonatal therapy.

Nursing and NTs alike require highly specialized knowledge for work in the labor-intensive NICU, as newborns are totally dependent and require complex technologic and personnel support for even routine functions such as breathing, thermoregulation, and feeding [53]. Interdisciplinary collaboration affects patient outcomes as trust and respect enhance communication about patient issues [54]. A staffing plan that optimizes each staff member’s ability to practice at the top of his or her skill level will lead to quality patient care and fiscal responsibility [45, p. 28]. As such, the high specialization of NICU nursing usually precludes nursing floats from other hospital units during times of higher census [53]. In kind, it is recommended that NTs be assigned to the NICU with dedicated days without cross coverage to other units (e.g., adult inpatient, adult acute care, pediatrics, etc.) or, at a minimum, avoidance of same-day cross-unit work.

### Proposed formula for determining staff allocations for Levels III and IV NICUs

The preponderance of evidence establishing the relationship between staffing and quality patient outcomes is currently recognized by many. However, there is marked variability across units and states in staffing of NT and limited resources to inform managers and administrators on how to allocate NT efficiently. Staffing model complexities include variables such as unit function variances and organization-related variances to name a few [45, p. 26–27]. These variables allow for consideration of staff participation and involvement in quality measurement activities, development of critical pathways or protocols, evaluation of practice outcomes, collaboration with other staff, and opportunity for care coordination. Given the differences of patient needs across different levels of NICUs, patient acuity levels should factor into NT staffing measures, with the goal of preventing poor outcomes. Specific data analyzing the adequacy of NICU staffing are scarce; however, recommendations for considering higher staffing with higher acuity are suggested [47].

Patient experience and NICU-related outcomes are associated with both direct and indirect therapy services of NTs. Indirect services are essential to the preventative/habilitative model of care and are often overlooked in NT staffing as therapists are most typically staffed using a rehabilitative lens. Examples of NT services that contribute to infant neurodevelopmental outcomes, staff support, and psychosocial support of families include, staff/family education regarding positioning and handling, management of environmental factors, participation in unit-wide program development, and preparation of the family for discharge and follow-up services. Given outcomes related to neuroprotective care utilizing a preventative/habilitative model, NT staffing should allow for a minimum of 25% nonbillable time for the continuous quality improvement activities that address these infants’ unique and complex issues.

Although staffing calculations that include both (1) worked hour per unit of service (WHPUOS) and (2) hours per patient day (HPPD) are commonly utilized for hospital staffing of rehabilitation departments, the preventive/habilitative model of care of the NICU necessitates a different formula for staffing NTs. Staffing must include allotment for direct and indirect services (billable and nonbillable care) to include collaborative care with MD/RN/RT/parents, planning, staff support/education/mentoring, bedside rounds, discharge planning, parent education/support, and NBN consultation. Table 3 presents calculations for recommended minimum baseline staffing of elemental NTs in the Levels III and IV NICU (including Level II when associated with Level III or IV units).

**Table 3:** Staffing calculation for recommended minimum neonatal therapy FTEs.

**Step 1**. Potential NT referrals equal 90 percent of the annualized average daily census (i.e., coverage needed to ensure adequate staffing to address requisite direct and indirect NT services): $\left[ {{\rm{Annualized}}\;{\rm{average}}\;{\rm{daily}}\;{\rm{census}}} \right]\times 0.90 = [{\rm{number}}\;{\rm{of}}\;{\rm{potential}}\; {\rm{referrals}}]$	
**Step 2**. FTE staffing calculation is determined by dividing the number of potential referrals by 13–15 infants to reflect the average patient coverage per NT. This identifies the number of FTEs needed with an integrated model in Level III or IV NICUs:	
$$\frac{{\rm{Potential}}\;{\rm{referrals}}}{{13 - 15({\rm{infants}}\left[ {\rm{range}}\,{\rm{to}}\,{\rm{allow}}\,{\rm{for}}\,{\rm{higher}}\,{\rm{acuity}} \right])}} = {\rm{FTEs}}$$	
**Examples:**	Level III/IV moderate acuity:
	$$\frac{{60 \, \times \, 0.90 \, = \, 54}}{{15}} = 3.6 \, {\rm{FTEs}}$$
	Level III/IV high acuity:
	$$\frac{{60 \, \times \, 0.90 \, = \, 54}}{{13}} = 4.1 \, {\rm{FTEs}}$$

For hospitals where services are needed that include research, weekend coverage, higher acuity, or increased NT presence, an adjustment to the formula in Step 2 is recommended (i.e., divisor of 12–15). This formula, when used to determine adequate staffing of NTs, supports the provision of quality care that facilitates optimal development and support of infants and their families in the NICU.

### Path to acquire neonatal therapy expertize

The clinical and professional reasoning that NTs bring to the NICU is based on the understanding of medical factors, human development, and the interplay between the environment and the sensory system. The AAP Committee on Fetus and Newborn and American College of Obstetricians and Gynecologists Committee on Obstetric Practice 2017 Perinatal Guidelines (AAP and ACOG), eighth edition, recommends that services of at least one OT or PT with neonatal expertize in addition to a professional with neonatal feeding expertize be available in Levels III and IV NICUs [1]. Table 4 highlights a recommended process for gaining the specialized knowledge, skills and abilities needed to demonstrate neonatal expertize as a therapist.

**Table 4:** Recommended preparation components for practice as a neonatal therapist.

• Minimum of 3 years of experience as a practicing OT, PT, or SLP in a pediatric practice setting is highly recommended
• Specialized mentoring in neonatal therapy (in-person and/or online)
• Initial and ongoing participation in peer-reviewed education specific to neonatal therapy is necessary for safe and effective practice
• Alignment with relevant professional organization
• Mentored practice hours and established competence in the NICU (before practicing independently)
• Within 2 years of completing 3500 h of direct practice in NICU, pursuing neonatal therapist certification is recommended

The AOTA, APTA, and ASHA organizations have developed discipline-specific NICU competency resources to support the professional development of their membership. In 2018, the AOTA Practice Council reauthorized a description of the role of OTs in the NICU [20]. The APTA Academy of Pediatric Physical Therapy Neonatal Task Force published revised NICU competency recommendations in 2009 and 2010 [21, 22]. In addition, APTA has endorsed three American Board of Physical Therapy Residency and Fellowship Education (ABPTRFE) accredited neonatology fellowship programs designed to prepare licensed professionals within the physical therapy discipline to gain the skills needed for advanced clinical competency in neonatal physical therapy [55, 56]. ASHA has published multiple practice policies on the organization website to provide the membership with needed information related to clinical and instrumental assessment of oral motor/feeding capacities applicable to SLP working in the NICU setting [23, 57–60]. NANT has published documents defining the common roles and Core Scope^©^ of practice of NTs [41, 42]. Despite these discipline-specific and discipline-independent resources designed to delineate the knowledge, skills and abilities needed by NTs, there is currently no discipline-specific board certification examination process in place to validate acquisition of these skills. In compliance with criteria established by the Institute for Credentialing Excellence, a national independently governed multidisciplinary board was established in 2015 to oversee the development and administration of a neonatal certification process to validate and standardize the requisite experience, education, and knowledge needed for therapists to work in the NICU setting. Comprised of experienced NTs from across the USA and around the world representing the disciplines of occupational therapy, physical therapy, or speech-language pathology, the NTNCB established criteria for specialty neonatal certification consistent with AOTA, APTA, and ASHA recommendations designed to ensure a minimum standard for the provision of safe and competent care to high risk infants and their families in the NICU [61]. Licensed therapists who possess the necessary knowledge and experience to function independently as a NT in the NICU may consider pursuing board certification to be awarded the designation of Certified Neonatal Therapist (CNT).

Upon completion of prerequisite competency requirements and successfully passing a written examination, the NTNCB awards the specialty designation of CNT to duly licensed OT, PT, or SLP who have demonstrated evidence that they have attained the specialized professional qualifications needed to provide competent neonatal therapeutic services in the NICU. The CNT designation applies only to NTs who maintain current licensure/credentialing or permission to practice as a therapist under the jurisdiction of the respective licensing boards for OT, PT, and SLP. The NTNCB neonatal certification process is endorsed by the NANT [61]. The prerequisite competencies required to apply for the NTNCB certification examination are summarized in Table 5 [61].

**Table 5:** Prerequisite requirements for NTNCB Certified Neonatal Therapist (CNT) designation.

NTNCB Certified Neonatal Therapist requirements^a^
• PT, OT, or SLP credentialing for a period of 3 years
• 3500 h of direct practice in the NICU
• 40 h of education related to NICU practice within the past 3 years
• 40 h of mentored experiences
• Passing score on the Neonatal Therapy National Certification Exam

^**a**^Neonatal therapy national certification information may be accessed at: https://www.ntncb.com.

## Summary/conclusions

A rapidly expanding body of evidence supports the improved scope of outcomes for all involved stakeholders when a comprehensive neuroprotective developmental care model is applied in the NICU setting. Recommendations include neonatal therapy expertize as essential for optimal delivery of an integrated family-centered neuroprotective care model. Provision of therapy services in the NICU is an advanced area of practice for OT, PT, and SLP that requires specialized knowledge and experience to function independently as an expert NT in the NICU. Recommended preparation resources are available to assist licensed professionals to acquire the discipline-specific expertize needed to meet practice standards in this acute medical practice setting. It is incumbent on the individual therapist to work collaboratively within a transdisciplinary service delivery model to maximize the effectiveness of services of all care providers while simultaneously working to gain the requisite discipline-specific advanced training needed to fulfill the unique contributions the respective disciplines offer in this complex acute care setting.

## References

[1] American Academy of Pediatrics. Guidelines for Perinatal Care/American Academy of Pediatrics (and) the American College of Obstetricians and Gynecologists 8th ed. Pediatrics. American Academy of Pediatrics; Washington, D.C. 2017; ISBN: 978-1610020879 AAP.

[2] Butruille L, Blouin A, De Jonckheere J, Mur S, Margez T, Rakza T (2017). Impact of skin-to-skin contact on the autonomic nervous system in the preterm infant and his mother. Infant Behav Dev..

[3] Charpak N, Tessier R, Ruiz J, Hernandez J, Uriza F, Villegas J, et al. Twenty-year follow-up of kangaroo mother care versus traditional care. Pediatrics. 2017;139:2016–63. 10.1542/peds.2016-2063 27965377

[4] Macho P (2017). Individualized developmental care in the NICU: a concept analysis. Adv Neonatal Care..

[5] Montirosso R, Giusti L, De Carli P, Tronick E, Borgatti R (2018). Developmental care, neonatal behavior and postnatal maternal depressive symptomatology predict internalizing problems at 18 months for very preterm children. J Perinatol.

[6] Qiu J (2017). Effect of combined music and touch intervention on pain response and beta-endorphin and cortisol concentrations in late preterm infants. BMC Pediatr..

[7] Symington A, Pinelli J. Developmental care for promoting development and preventing morbidity in preterm infants. Cochrane Database Syst Rev. 2006;2:CD001814. 10.1002/14651858.CD001814.pub2 PMC8962209 16625548

[8] Welch MG (2017). Family nurture intervention in preterm infants increases early development of cortical activity and independence of regional power trajectories. Acta Paediatr..

[9] Yen-Ting Y (2017). Family-centered care improved neonatal medical and neurobehavioral outcomes in preterm infants: randomized controlled trial. Phys Ther.

[10] Milette I, Martel MJ, Ribeiro da Silva M, Coughlin-McNeil M (2017). Guidelines for the institutional implementation of developmental neuroprotective care in the NICU. Part B: recommendations and justification. A joint position statement from the CANN, CAPWHN, NANN and COINN. Can J Nurs Res.

[11] Milette I, Martel MJ, Ribeiro da Silva M, Coughlin-McNeil M (2017). Guidelines for the institutional implementation of developmental neuroprotective care in the NICU. Part A: background and rationale. A joint position statement from the CANN, CAPWHN, NANN and COINN. Can J Nurs Res.

[12] National Association of Neonatal Therapists. Neonatal therapy core scope of practice. 2014. https://neonataltherapists.com/resources/. Accessed 3 Nov 2019.

[13] Sturdivant C (2013). A collaborative approach to defining neonatal therapy. Newborn Infant Nurs Rev..

[14] Aita M, Snider L (2003). The art of developmental care in the NICU: a concept analysis. J Adv Nurs..

[15] Craig J, Glick C, Phillips R, Hall S, Smith J, Browne J (2015). Recommendations for involving the family in developmental care of the NICU baby. J Perinatol..

[16] Kinner MD, Browne JV (1997). Developmental care in advanced practice neonatal nursing education. J Nurs Educ.

[17] Ross K, Heiny E, Conner S, Spener P, Pineda R (2017). Occupational therapy, physical therapy and speech-language pathology in the neonatal intensive care unit: patterns of therapy usage in a level IV NICU. Res Dev Disabil.

[18] Coughlin M. Age-appropriate care of the prematures and critically ill hospitalized infant: NANN guidelines for practice. Glenview, IL: National Association of Neonatal Nurses; 2011.

[19] Coughlin M, Gibbins S, Hoath S (2009). Core measures for developmentally supportive care in NICU: theory, precedence and practice. J Adv Nurs.

[20] Craig J, Carroll S, Ludwig S, Sturdivant C (2018). Occupational therapy’s role in the neonatal intensive care unit. Am J Occup Ther..

[21] Sweeney JK, Heriza CB, Blanchard Y (2009). Neonatal physical therapy. Part 1: clinical competencies and neonatal intensive care unit clinical training models. Pediatr Phys Ther.

[22] Sweeney JK, Heriza CB, Blanchard Y, Dusing S (2010). Neonatal physical therapy. Part 2: practice frameworks and evidence-based practice guidelines. Pediatr Phys Ther..

[23] Ad Hoc Committee on Speech-Language Pathology Practice in the Neonatal Intensive Care Unit (NICU). Knowledge and skills needed by speech-language pathologists providing services to infants and families in the NICU environment. American Speech-Language Hearing Association [ASHA Practice Policy]. 2004. https://www.asha.org/policy/KS2004-00080/. Accessed 11 Mar 2019.

[24] Álvarez MJ, Fernández D, Gómez-Salgado J, Rodríguez-González D, Rosón M, Lapeña S (2017). The effects of massage therapy in hospitalized preterm neonates: a systematic review. Int J Nurs Stud.

[25] Dusing SC, Brown SE, Van Drew CM, Thacker LR, Hendricks-Munoz KD (2015). Supporting play exploration and early development intervention from NICU to home: a feasibility study. Pediatr Phys Ther.

[26] Dusing SC, Tripathi T, Marcinowski EC, Thacker LR, Brown LF, Hendricks-Munoz KD (2018). Supporting play exploration and early developmental intervention versus usual care to enhance development outcomes during the transition from the neonatal intensive care unit to home: a pilot randomized controlled trial. BMC Pediatr..

[27] Milgrom J, Newnham C, Anderson PJ, Doyle LW, Gemmill AW (2010). Early sensitivity training for parents of preterm infants: impact on the developing brain. Pediatr Res..

[28] Finch-Edmondson M, Morgan C, Hunt RW, Novak I (2019). Emergent prophylactic, reparative and restorative brain interventions for infants born preterm with cerebral palsy. Front Physiol..

[29] Robinson LD (2003). An organizational guide for an effective developmental program in the NICU. J Obstet Gynecol Neonatal Nurs.

[30] Altimier L, Kenner CM, Damus K (2015). The wee care neuroprotective NICU program (Wee Care): the effect of a comprehensive developmental care training program on seven neuroprotective core measures for family-centered developmental care of premature neonates. Newborn Infant Nurs Rev.

[31] Broom M, Parsons G, Carlisle H, Kecskes Z, Thibeau S (2017). Exploring parental and staff perceptions of the family-integrated care model: a qualitative focus group study. Adv Neonatal Care..

[32] Cardin AD, Rens L, Stewart S, Danner-Bowman K, McCarley R, Kopsas R (2015). Neuroprotective core measures 1–7: a developmental care journey: transformations in NICU design and caregiving attitudes. Newborn Infant Nurs Rev.

[33] Moody C, Callahan TJ, Aldrich H, Gance-Cleveland B, Sables-Baus S (2017). Early initiation of newborn individualized developmental care and assessment program (NIDCAP) reduces length of stay: a quality improvement project. J Pediatr Nurs..

[34] Montirosso R, Giusti L, De Carli P, Tronick E, Borgatti R (2018). Developmental care, neonatal behavior and postnatal maternal depressive symptomatology predict internalizing problems at 18 months for very preterm children. J Perinatol.

[35] Ohlsson A, Jacobs SE (2013). NIDCAP: a systematic review and meta-analyses of randomized controlled trials. Pediatrics..

[36] Ustad T, Evensen KA, Campbell SK, Girolami GL, Helbostad J, Jorgensen L (2016). Early parent-administered physical therapy for preterm infants: a randomized controlled trial. Pediatrics..

[37] Gibbins S, Hoath SB, Coughlin M, Gibbins A, Franck L (2008). The universe of developmental care: a new conceptual model for application in the NICU. Adv Neonatal Care..

[38] Als H (1986). A synactive model of neonatal behavioral organization. Phys Occup Ther Pediatr.

[39] Gibbins S, Hoath SB, Coughlin M, Gibbins A, Franck L (2008). The universe of developmental care: a new conceptual model for application in the NICU. Adv Neonatal Care..

[40] Miyagishima S, Himuro N, Kozuka N, Mori M, Tsutsumi H (2017). Family-centered care for preterm infants: parent and physical therapist perceptions. Pediatrics..

[41] National Association of Neonatal Therapists. Neonatal therapy core scope of practice. 2014. https://neonataltherapists.com/resources/. Accessed 11 Mar 2019.

[42] Ludwig S, Sturdivant C. Neonatal therapist job description. 2011. https://neonataltherapists.com/resources/. Accessed 11 Mar 2019.

[43] Mefford LC, Alligokod MR (2011). Evaluating nurse staffing patterns and neonatal intensive care unit outcomes using Levine’s conservation model of nursing. J Nurs Manag.

[44] Dal Molin A, Gatta C, Gilor CB, Ferrua R, Cena T, Manthey M (2018). The impact of primary nursing care pattern: results from a before-after study. J Clin Nurs.

[45] Hunt P. Developing a staffing plan to meet inpatient unit needs. Nursing management. Wolters Kluwer Health, Inc. 2018. 25-31. www.nursingmanagement.com. Accessed 11 Mar 2019. 10.1097/01.NUMA.0000532326.62369.9b 29629976

[46] Atwater A, Hartmann E, Brown B, Carteaux P, Freeman M (2006). Evaluation and development of potentially better practices for staffing in neonatal intensive care units. Pediatrics..

[47] National Association of Neonatal Nurses. RN staffing in the neonatal intensive care unit: position statement #3061. National Association of Neonatal Nurses; 2014.

[48] American Nurses Association. (2012). ANA’s principles for nurse staffing.

[49] Ross K, Heiny E, Conner S, Spener P, Pineda R (2017). Occupational therapy, physical therapy and speech-language pathology in the neonatal intensive care unit: patterns of therapy usage in a Level IV NICU. Res Dev Disabil.

[50] Rothberg M, Abraham I, Lindenauer P, Rose D (2015). Improving nurse-to-patient staffing ratios as a cost-effective safety intervention. Med Care..

[51] Needleman J, Buerhaus P, Mattke S, Stewart M, Zelevinsky K (2002). Nurse-staffing levels and the quality of care in hospitals. N Engl J Med.

[52] Rogowski J, Staiger D, Patrick T, Harbar J, Kenny M, Lake E (2013). Nurse staffing and NICU infection rates. JAMA Pediatr.

[53] Richardson D, Zupancic J, Escobar G, Ogino M, Pursley D, Mugford M (2001). A critical review of cost reduction in neonatal intensive care I. The structure of costs. J Perinatol..

[54] Pollack MM, Koch MA (2003). Association of outcomes with organizational characteristics of neonatal intensive care units. Crit Care Med.

[55] American Board of Physical Therapy Residence and Fellowship Education (ABPTRFE) credentialed fellowship programs in physical therapy website. 2018. http://www.abptrfe.org/ForParticipants/AboutFellowships/. Accessed 11 Mar 2019.

[56] Keithley R, Boynewicz K, Delapp S, Pineda I. APTA Pediatric Academy Resource Guide for Physical Therapy Practice in the NICU. https://pediatricapta.org/special-interest-groups/NN/pdfs/Neonatal%20Didactic%20Training%20Resource%20list.pdf. Accessed 11 Mar 2019.

[57] Dysphagia Document Review and Revision Working Group. Knowledge and skills needed by speech-language pathologists providing services to individuals with swallowing and/or feeding disorders. Online: American Speech-Language Hearing Association [ASHA Practice Policy]. 2002. https://www.asha.org/policy/KS2002-00079/. Accessed 11 Mar 2019.

[58] ASHA Special Interest Division 13, Swallowing and Swallowing disorders (Dysphagia). Knowledge and skills needed by speech-language pathologists performing endoscopic assessment of swallowing functions. Online: American Speech-Language Hearing Association [ASHA Practice Policy]. 2002. https://www.asha.org/policy/KS2002-000.69.htm. Accessed 11 Mar 2019.

[59] ASHA Special Interest Division 13, Swallowing and Swallowing disorders. Knowledge and skills needed by speech-language pathologists performing videofluoroscopic swallowing studies. Online: American Speech-Language Hearing Association [ASHA Practice Policy]. 2004. https://www.asha.org/policy/KS2004-00076/. Accessed 11 Mar 2019.

[60] ASHA Working Group on Endoscopy. Use of endoscopy by speech-language pathologists: Position statement. Online: American Speech-Language Hearing Association [ASHA Practice Policy]. 2008. https://www.asha.org/policy/PS2008-00297/. Accessed 11 Mar 2019.

[61] Neonatal Therapy National Certification Board website. 2015. https://ntncb.com/. Accessed 11 Mar 2019.

